# Enhancement of Biocompatibility of High-Transparency Zirconia Abutments with Human Gingival Fibroblasts via Cold Atmospheric Plasma Treatment: An In Vitro Study

**DOI:** 10.3390/jfb15070200

**Published:** 2024-07-21

**Authors:** Miao Zheng, Xinrong Ma, Jianguo Tan, Hengxin Zhao, Yang Yang, Xinyi Ye, Mingyue Liu, Heping Li

**Affiliations:** 1 Department of Stomatology, Peking University Third Hospital, Beijing 100191, China; zhengmiao@bjmu.edu.cn (M.Z.); maxinrong95@bjmu.edu.cn (X.M.); 2 Department of Prosthodontics, Peking University School and Hospital of Stomatology and National Center for Stomatology and National Clinical Research Center for Oral Diseases and National Engineering Research Center of Oral Biomaterials and Digital Medical Devices, Beijing 100081, China; kqtanjg@bjmu.edu.cn (J.T.); yydavid@pku.edu.cn (Y.Y.); yexinyi@bjmu.edu.cn (X.Y.); 3 Department of Engineering Physics, Tsinghua University, Beijing 100084, China; zhaohx22@mails.tsinghua.edu.cn; 4 First Clinical Division, Peking University School and Hospital of Stomatology, Beijing 100034, China

**Keywords:** cold atmosphere plasma, high-transparency zirconia, abutment surface modification, reactive oxygen species

## Abstract

The objective of this study was to explore the effects of cold atmospheric plasma (CAP) treatment on the biological behavior of human gingival fibroblasts (HGFs) cultured on the surface of high-transparency zirconia. Two types of zirconia, 3Y-ZTP and 4Y-PSZ, were subjected to a CAP treatment for various treatment durations. Analyses of the physical and chemical properties of 3Y-ZTP and 4Y-PSZ were conducted using scanning electron microscopy, contact angle measurements, and X-ray photoelectron spectroscopy, both before and after CAP treatment. The biological responses of HGFs on both surfaces were assessed using CCK-8 assay, confocal laser scanning microscopy, and real-time PCR. Initially, the oxygen and hydroxyl contents on the surface of 4Y-PSZ exceeded those on 3Y-ZTP. CAP treatment enhanced the surface hydrophilicity and the reactive oxygen species (ROS) content of 4Y-PSZ, while not altering the surface morphology. After CAP treatment, HGFs’ adhesion on 4Y-PSZ was superior, with more pronounced effects compared to 3Y-ZTP. Notably, HGFs counts and the expression of adhesion-related genes on 4Y-PSZ peaked following the CAP exposures for 30 s and 60 s. Consequently, this study demonstrates that, following identical CAP treatments, 4Y-PSZ is more effective in promoting HGFs adhesion compared to traditional 3Y-ZTP zirconia.

## 1. Introduction

Implants have become the preferred choice for patients with defected dentition [1]. As the annual number of implant placements increases, biological complications, primarily peri-implant diseases, have emerged as significant factors compromising the long-term success of implant restoration [2]. Peri-implant soft tissue attachments, comprising epithelium and connective tissue, exhibit vulnerabilities. Specifically, a deficiency of hemidesmosomes in the peri-implant epithelium (PIE) and the absence of vascular and nerve distribution in the connective tissue are primary weaknesses leading to peri-implant diseases [3,4]. Enhancing the soft tissue bond between the implant abutment and the surrounding gingival is crucial for reducing the incidence of peri-implant diseases and enhancing the success rate of implant restorations [5].

Upon integration with the implant, the biocompatibility of the abutment significantly influences the attachment of the adjacent periodontal tissue [6]. While titanium has been the predominant material for implants and abutments over recent decades, its dark gray appearance significantly limits its use in anterior teeth [7,8,9]. Zirconia, an all-ceramic material, combines high strength and aesthetic appeal, making it the material of choice for many prosthetic applications [10]. Zirconia has consistently exhibited superior biocompatibility, low plaque retention, and favorable radiopacity in numerous in vivo and in vitro studies [11,12,13,14]. These attributes have progressively established it as the preferred abutment material for aesthetic implant restoration in anterior teeth [15]. 

Traditional zirconia, which includes yttrium oxide components (3Y-ZTP, 3%mol yttrium oxide), presents as white and relatively opaque. Responding to increased aesthetic demands and the need for higher transparency, a new generation of zirconia known as high-transparency zirconia (4Y-PSZ) has been developed. This material contains 4% mol yttrium oxide and a greater proportion of cubic phases, enhancing its transparency and decreasing its opacity [16,17]. The aesthetic qualities and superior transparency of 4Y-PSZ are notably improved over traditional zirconia, making it suitable for anterior crowns and abutments.

To enhance early adhesion and long-term stability between high-transparency zirconia abutments and surrounding gingiva, various surface modification techniques have been developed [18]. Research has predominantly shown that the smooth, polished surface of traditional zirconia facilitates the adhesion and proliferation of fibroblasts [19,20,21]. Wettability and surface energy are critical properties influencing biocompatibility. UV-treated zirconia surfaces exhibit increased wettability, which supports the attachment of proteins and other macromolecules, thereby enhancing cell adhesion, spreading, proliferation, and differentiation [22,23,24]. Bionic surface coatings, including hydroxyapatite (HA), β-tricalcium phosphate (β-TCP), collagen, and chitosan, are recognized for their potential to promote soft tissue formation around zirconia abutments [25,26]. However, photofunctionalization and biofunctionalization are time-consuming and complex processes. Moreover, research on surface modification of high-transparency zirconia abutments is limited. Thus, there is a pressing need to identify a surface treatment method that is simple to implement and can enhance the wettability of high-transparency zirconia without altering its surface roughness.

Plasma treatment has been demonstrated to improve implant osseointegration and soft tissue integration by increasing the surface energy of implants and abutments [27,28]. High-intensity, long-duration cold plasma treatment releases substantial amounts of reactive oxygen species (ROS) and reactive nitrogen species (RNS) [29,30,31]. The range of oxygen-based species produced by plasmas includes hydroxyl (OH), hydrogen peroxide (H_2_O_2_), superoxide (O_2_^−^•), hydroxyl radical (•OH), singlet oxygen (^1^O_2_), and ozone (O_3_) [32]. ROS have been shown to promote stem cell differentiation and cell proliferation [33]. Our previous research has demonstrated that cold atmospheric plasma (CAP) treatment of traditional zirconia (3Y-ZTP) abutment is beneficial for the adhesion and proliferation of human gingival fibroblasts (HGFs), and that the surface activation of zirconia after plasma treatment is stoichiometry-dependent [34,35]. Conversely, 4Y-PSZ, with a higher concentration of yttrium oxide, may display altered surface properties and biocompatibility under the same CAP treatment conditions. The aim of this study is to further investigate the impact of CAP treatment on 4Y-PSZ to uncover the potential applications of CAP in high-transparency zirconia. Additionally, this study will clarify the effects of ROS levels on HGFs attachment. The null hypotheses are that CAP treatment does not improve HGFs’ adhesion to high-transparency zirconia and that there is no measurable relationship between the ROS generated by CAP treatment and the biological responses of HGFs. 

## 2. Materials and Methods

### 2.1. Preparation of Zirconia Disks

The two zirconia materials used in this experiment were both sintered at high temperatures. The content of the Y_2_O_3_ stabilizer added during the production process varied: 3Y-ZTP contained 3% yttrium oxide (Mol%), while 4Y-PSZ contained 4% yttrium oxide (Mol%). Consequently, the crystal phase structures of the two zirconia materials differed. 3Y-ZTP exhibited a tetragonal phase structure, while 4Y-PSZ was composed of 75% tetragonal phase and 25% cubic phase.

Zirconia disks, 3Y-ZTP and 4Y-PSZ (Upcera, Shenyang, China), each 20 mm in diameter and 2 mm in thickness, were prepared. The crystallographic structure of the zirconia conformed to the properties of zirconium yttrium oxide. These disks were shaped using a computer-aided design and manufacturing (CAD-CAM) process. All specimens were serially polished with a water coolant to 2000 grit, then ultrasonically cleaned in absolute ethanol and distilled water for 20 min, air-dried, and stored at room temperature (25 °C) prior to surface treatment. The 3Y-ZTP and 4Y-PSZ disks were each divided into four groups based on CAP treatment duration. The experimental groups received CAP treatments of 30 s, 60 s, and 90 s, while the control groups underwent no plasma treatment for comparison. 

### 2.2. Helium CAP Jet

The plasma generator utilized in this experiment was supplied by the Plasma Health Scientech Group (PHSG), of Tsinghua University. Detailed schematic diagrams of the device are available in previous studies [35,36]. High-purity helium was used to generate plasma at a flow rate of 10.8 standard liters per minute (slpm). A uniform glow discharge was achieved in a fixed volume of 25.25 cm^3^, with power densities ranging from 0.36 to 0.44 W/cm^3^ and applied voltages between 4.64 and 4.72 kV at a driving frequency of 26 kHz. The zirconia disk was positioned perpendicularly to the plasma generator, 1.0 cm away on the objective table. Each disk underwent treatment for durations of 30 s, 60 s, and 90 s, corresponding to the various experimental groups.

### 2.3. Surface Roughness

Surface roughness was assessed using a Mitutoyo Surftest 401 Analyzer Series 200 (Mitutoyo Corp., Minatoku, Japan). The cut-off value of the device was set to 0.8 mm and the measuring length was 4 mm. Five measurements were taken at various points on each disk. The final reported arithmetical mean surface roughness (Ra in μm) represented the average across five disks.

### 2.4. Surface Wettability

Surface wettability was evaluated using a contact angle meter (SL200, Kino Industry, Norcross, GA, USA). Measurements were conducted at three distinct points on each disk, 3 s after applying a 1 μL water droplet. Four disks in each group were tested at these three points and the average resulting from these measurements was recorded as the wettability of each group.

### 2.5. XPS Analysis

X-ray photoelectron spectroscopy (XPS) (ESCALAB 250, Thermo Fisher Scientific, Waltham, MA, USA) was utilized to quantitatively analyze the atomic composition of zirconia, focusing on carbon and oxygen elements. Survey spectra were acquired at a pass energy of 200 eV and detailed spectra for the C 1s and O 1s peaks were recorded at 30 eV pass energy. All the spectra were normalized by fixing the C 1s photoemission peak (284.8 eV) to the same value to facilitate comparison between samples.

### 2.6. Cell Culture

Human gingival fibroblasts (HGFs) obtained from Procell Life Science and Technology (Wuhan, China) were cultured in Dulbecco’s Modified Eagle’s Medium (Gibco BRL Co., Gaithersburg, MD, USA) supplemented with 10% fetal bovine serum and 1% antibiotic–antimycotic solution. The cells were maintained at 37 °C in a 95% humidity and 5% CO_2_ incubator. Confluent cells were subcultured using trypsinization, with cells from the fourth-to-seventh passages being selected for experiments. 

### 2.7. Cell Adhesion and Proliferation Assays (CCK-8 Analysis)

Zirconia disks were placed into 24-well plates using sterilized forceps and HGFs were seeded at a density of 1.0 × 10^5^/zirconia disk (*n* = 5 for each group). Initial adhesion occurred over 30 min before adding 1 mL of complete medium to each well. Cultures were maintained for durations of 3, 24, 48, and 72 h. Cell adhesion and proliferation were assessed using a Cell Counting Kit-8 (CCK-8, Dojindo, Kyushu, Japan). After specified incubation periods, HGFs were washed thrice with phosphate-buffered saline (PBS) before adding 100 μL of CCK-8 solution per mL of medium. After 2 h of incubation at 37 °C, the optical density at 450 nm was measured using a spectrophotometer (ELX808, BioTek, Winooski, VT, USA). Experiments were conducted in triplicates.

### 2.8. Confocal Laser Scanning Microscopic Analysis

Indirect immunofluorescence (IIF) was conducted to assess the adhesion and proliferation of HGFs on zirconia disk surfaces. Cells numbering either 5 × 10^4^ or 1 × 10^4^ were seeded onto disks and incubated for either 3 h or 24 h, respectively. The attached cells were fixed in 4% paraformaldehyde, after the unattached cells were removed with sterile PBS. Subsequently, the samples were stained with fluorescein isothiocyanate–phalloidin (actin filaments in green, Sigma, St. Louis, MO, USA) and 4′6-diamidino-2-phenylindole (nuclei in blue, Roche, Basel, Switzerland). Images were captured using a confocal laser scanning microscope (LSM710, Zeiss, Germany). Cell perimeters and areas were quantified using an image analyzer (Image J, version 2, NIH).

### 2.9. Gene Expression Analysis

Real-time polymerase chain reaction (real-time PCR) was employed to evaluate the expression of adhesion-related genes, such as focal adhesion kinase (FAK), integrins β3, fibronectin (FN), and vinculin (VCL), at the mRNA level. After 24 h of culturing, mRNA from six disks per group was extracted using TRIzol (Invitrogen, Grand Island, NY, USA). Total RNA (2 μg) was converted to cDNA with the RevertAid First Strand cDNA Synthesis Kit (Thermo Scientific, Waltham, MA, USA). Real-time PCR was conducted using Takara SYBR premix Ex Taq (Takara Bio, Shiga, Japan) on an Applied Biosystems 7500 Real-Time PCR system (Foster City, CA, USA). Specific primers, listed in Table 1, were used. Relative mRNA expression levels were normalized to the housekeeping gene GAPDH and analyzed using the comparative ΔΔCT method.

### 2.10. Statistical Analysis

Data were expressed as means ± standard deviations and analyzed using statistical software (version 25, IBM, Armonk, NY, USA). A one-way ANOVA was utilized to assess significance between different 4Y-PSZ groups. An independent sample t-test was employed to compare the biological responses of cells on the surfaces of 3Y-ZTP and 4Y-PSZ under identical treatment conditions. Analyses were conducted at a significance level of α = 0.05.

## 3. Results

### 3.1. Surface Characteristics

#### 3.1.1. Surface Topography

**Figure 1:**
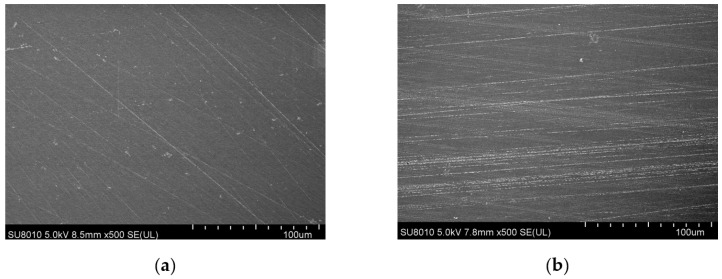
Surface morphology of 4Y-PSZ as SEM images. (**a**) 4Y-PSZ-0 s and (**b**) 4Y-PSZ-90 s (500 magnification).

**Figure 2:**
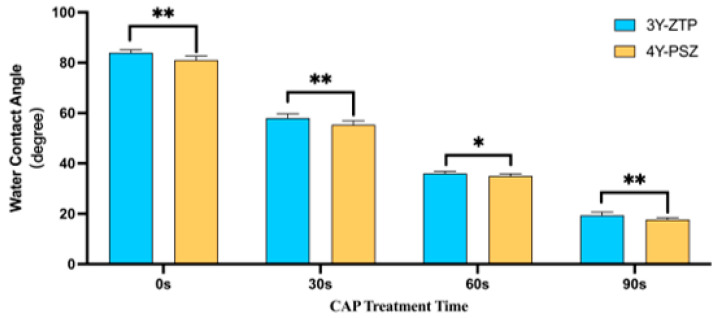
Surface wettability of 3Y-ZTP and 4Y-PSZ specimens at the same CAP treatment times (*n* = 12). (** *p* < 0.01; * *p* < 0.05).

4Y-PSZ disks exhibited a morphology characterized by typical ground marks and grooves in SEM images, and this morphology remained largely unchanged after CAP treatment (Figure 1). The average roughness (Ra) for both 3Y-ZTP and 4Y-PSZ was 0.05 ± 0.01 μm, with no significant changes in surface morphology or roughness post-treatment. Initially, the contact angles for zirconia were approximately 83.99 ± 1.22° (3Y-ZTP) and 81.04 ± 1.55° (4Y-PSZ). Following CAP treatment, there was a significant reduction in contact angles to 19.37 ± 1.36° (3Y-ZTP) and 17.70 ± 0.78° (4Y-PSZ), respectively (Table 2), indicating increasingly hydrophilic surfaces. The contact angle decreased proportionally with extended treatment duration. Notably, when treated for the same duration, 4Y-PSZ disks exhibited even lower contact angles compared to 3Y-ZTP disks (Figure 2).

#### 3.1.2. Surface Chemical Elemental Composition

**Figure 3:**
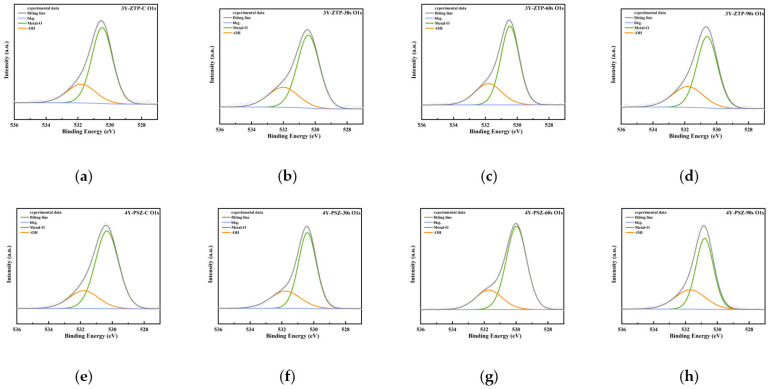
High-resolution XPS spectra of 3Y-ZTP and 4Y-PSZ surfaces. O 1 s high-resolution image of 3Y-ZTP and 4Y-PSZ. (**a**) 3Y-ZTP-0 s; (**b**) 3Y-ZTP-30 s; (**c**) 3Y-ZTP-60 s; (**d**) 3Y-ZTP-90 s; (**e**) 4Y-PSZ-0 s; (**f**) 4Y-PSZ-30 s; (**g**) 4Y-PSZ-60 s; and (**h**) 4Y-PSZ-90 s.

Peaks of C 1s, O 1s, Ca 2p3, N 1s, Y 3d, and Zr 3d were revealed in zirconia specimens by XPS analysis. Deconvolution of the spectrum indicated the presence of two types of oxidized Zr metal: Metal–O (B.E. = 530.48 ± 0.1 eV) and Hydroxide (B.E. = 531.79 ± 0.2 eV) (Figure 3). The atomic percentages of carbon (C at%) and oxygen (O at%) on the outermost surface of 3Y-ZTP and 4Y-PSZ, both before and after CAP treatment, are detailed in Table 3. Following CAP treatment, the peak of C at% decreased for both materials, as did the surface C/O ratio. CAP treatment also increased the incorporation of oxygen into zirconia surfaces, with the intensity of OH groups rising, particularly in the 4Y-PSZ-90 s group, which showed the highest OH content. Table 4 displays the percentage of OH groups following the deconvolution of oxygen element peaks.

### 3.2. Cell Adhesion and Proliferation Morphology

**Figure 4:**
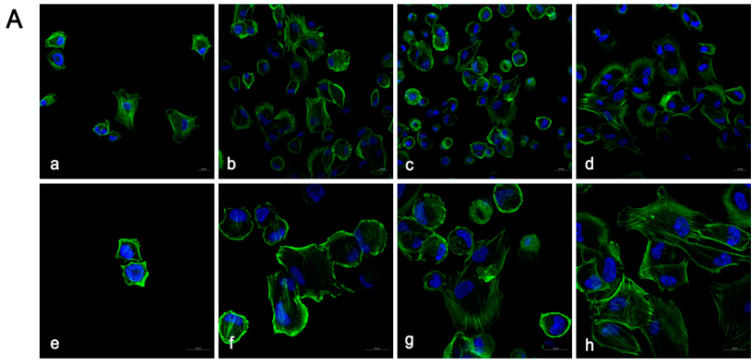

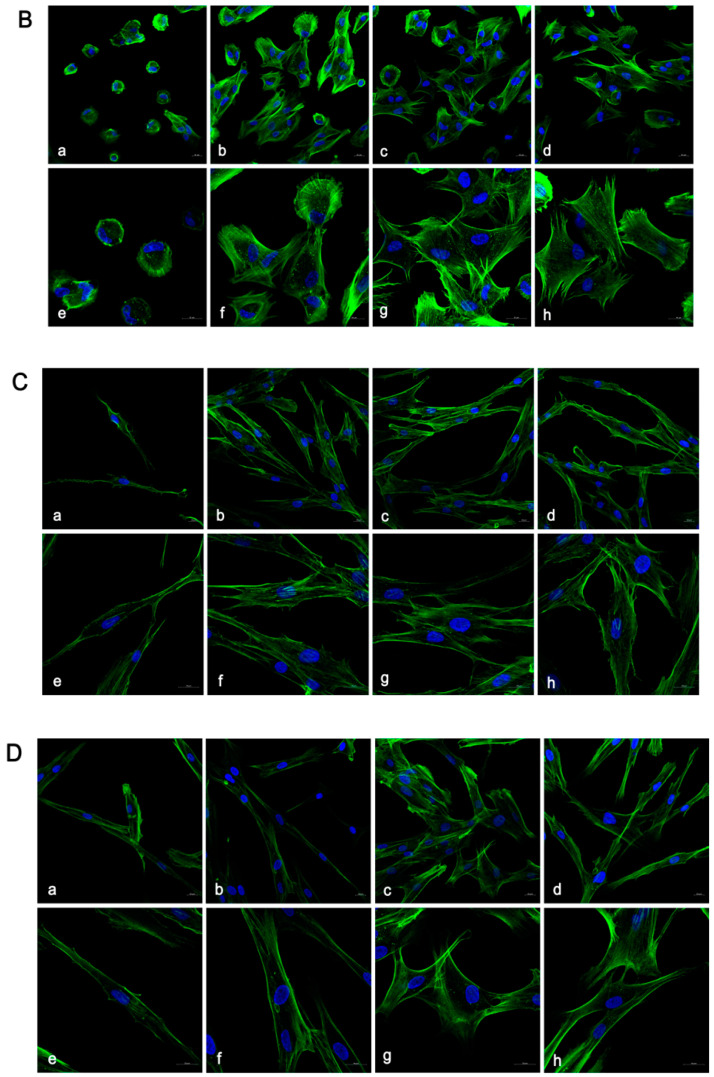
Observation of HGFs on 3Y-ZTP and 4Y-PSZ Surface by Confocal Laser Scanning Microscope. Confocal laser scanning microscopy images of HGFs on zirconia disks at 3 h (**A**,**B**) and 24 h (**C**,**D**) of culture. The CAP treatment times are 0 s (**a**,**e**), 30 s (**b**,**f**), 60 s (**c**,**g**), and 90 s (**d**,**h**). Low magnification (**a**–**d**), scale bar = 20 μm. High magnification (**e**–**h**), scale bar = 20 μm.

**Figure 5:**
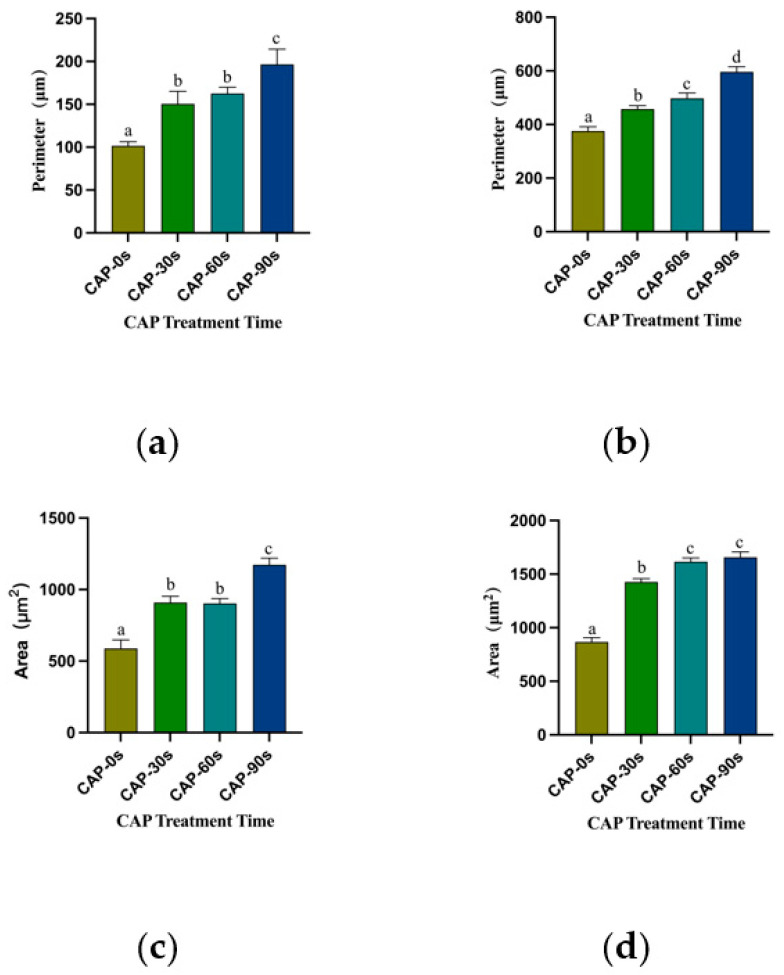
Perimeters and spreading areas of the HGFs on 4Y-PSZ surfaces. Perimeters (**a**,**b**) and spreading areas (**c**,**d**) of the HGFs on 4Y-PSZ surfaces at 3 h (**a**,**c**) and 24 h (**b**,**d**). Data are presented as means ± SD (n = 6). Identical letters indicate that the differences between those values have no sta-tistical significance (*p* > 0.05).

**Figure 6:**
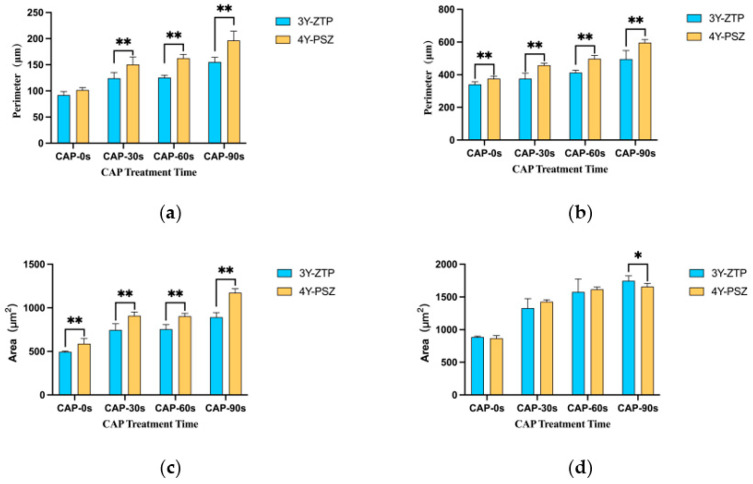
Comparison of HGFs’ perimeters and spreading areas on 3Y-ZTP and 4Y-PSZ surfaces at the same CAP treatment times. Perimeters (**a**,**b**) and spreading areas (**c**,**d**) of the HGFs on 3Y-ZTP and 4Y-PSZ surfaces at 3 h (**a**,**c**) and 24 h (**b**,**d**). Data are presented as means ± SD (*n* = 6). (** *p* < 0.01; * *p* < 0.05).

The critical stage for cell adhesion occurred 3 h post-inoculation, with both 3Y-ZTP and 4Y-PSZ demonstrating excellent biocompatibility, facilitating HGFs attachment. In both groups, HGFs cultured on the surfaces of both types of zirconia post-CAP treatment exhibited improved spreading, characterized by larger, green-stained cytoplasmic areas (Figure 4A,B). The perimeters and surficial areas of the HGFs in the three CAP treatment groups of 4Y-PSZ were significantly larger than those in the non-treatment group (*p* < 0.05) (Figure 5a,c). Following initial attachment, within the first 24 h, the morphology of HGFs transitioned from round to stringy. HGFs cultured on untreated zirconia displayed a narrow, elongated fusiform shape. Conversely, HGFs on 3Y-ZTP and 4Y-PSZ treated with CAP exhibited larger cytoplasmic areas, more pseudopodia, and intercellular interactions (Figure 4C,D). Statistical analysis confirmed that CAP treatment enhanced the spreading of HGFs on 4Y-PSZ surfaces (*p* < 0.05) (Figure 5b,d). Over time, the disparity in cell perimeters between the CAP-treated groups and the control group diminished, although differences in surface areas remained significant (Figure 5a–d). Comparisons of HGFs’ adhesion morphology on different materials indicated that cells displayed larger surface areas and perimeters on 4Y-PSZ compared to 3Y-ZTP, except for the 3 h perimeters and 24 h areas (*p* < 0.01). The disparity in material effects on cell morphology decreased over time (Figure 6).

### 3.3. Cell Adhesion and Proliferation Ability

**Figure 7:**
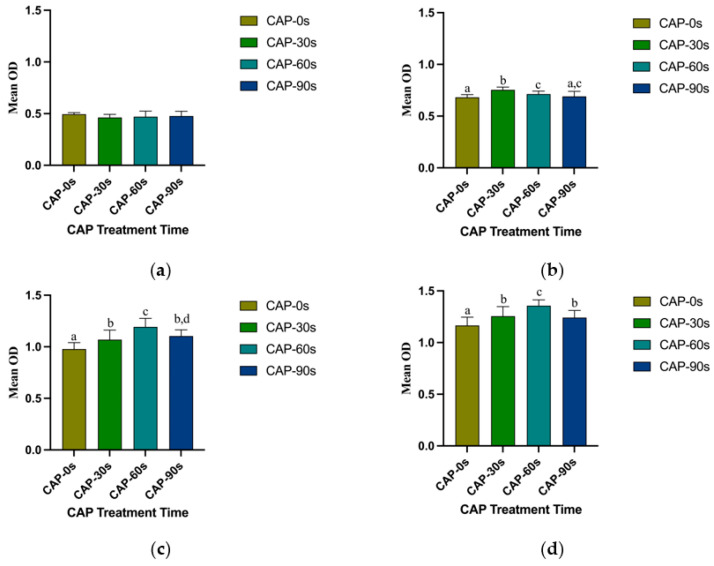
Quantitative measurements of the HGFs on 4Y-PSZ surfaces. HGFs’ attachment after culturing for (**a**) 3 h and (**b**) 24 h and proliferation after culturing for (**c**) 48 h and (**d**) 72 h. Data are presented as means ± SD (*n* = 12). Identical letters indicate that the differences between those values have no statistical significance (*p* > 0.05).

**Figure 8:**
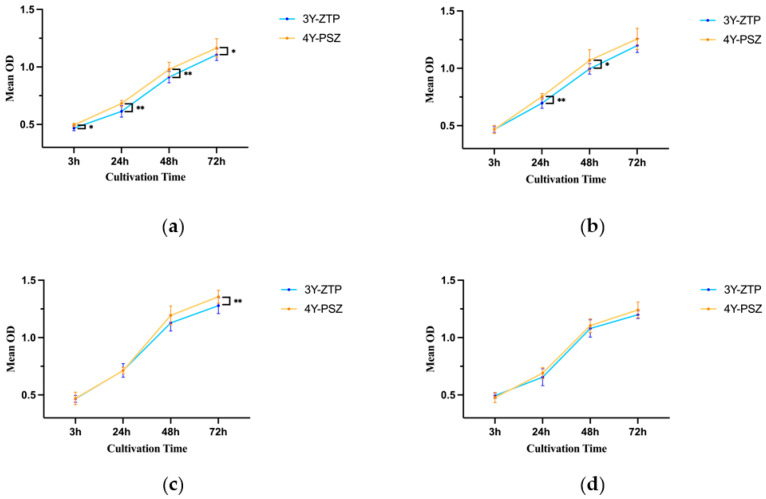
Quantitative comparison of HGFs on 3Y-ZTP and 4Y-PSZ surfaces at the same CAP treatment times (*n* = 12). CAP treatment times: (**a**) 0 s, (**b**) 30 s, (**c**) 60 s, and (**d**) 90 s (*p* > 0.05). (** *p* < 0.01; * *p* < 0.05).

The optical densities of HGFs attached to 4Y-PSZ surfaces after 24, 48, and 72 h of incubation were significantly higher on CAP-treated zirconia surfaces compared to those of the control group (*p* < 0.05) (Figure 7b–d). However, cell density showed no differences among the four groups after 3 h of culturing (Figure 7a). At 24 h, the CAP-30 s group exhibited the highest number of adhered HGFs, followed by the 60 s and 90 s groups (Figure 7b). Among the three CAP-treated groups, the 30 s and 60 s groups displayed a larger number of attached cells compared to the 90 s group at both 48 and 72 h (*p* < 0.05) (Figure 7c,d). With the increase of incubation time, the benefits of CAP treatment and the differences between treatment groups became more pronounced. Comparing the adhesion of HGFs on 3Y-ZTP and 4Y-PSZ surfaces, the 4Y-PSZ surface was more conducive to the adhesion and proliferation of HGFs than the 3Y-ZTP surfaces (*p* < 0.05) (Figure 8a). After 30 s of CAP treatment, the 4Y-PSZ surface demonstrated greater support for cell adhesion and proliferation at 24 and 48 h (*p* < 0.05) (Figure 8b). With extended CAP treatment durations, the 4Y-PSZ surface also showed improved support for HGFs’ biological behavior at different culturing times (30 s at 24 and 48 h; 60 s at 72 h) (*p* < 0.05) (Figure 8b–d).

### 3.4. Expression of Adhesion and Proliferation-Related Genes

**Figure 9:**
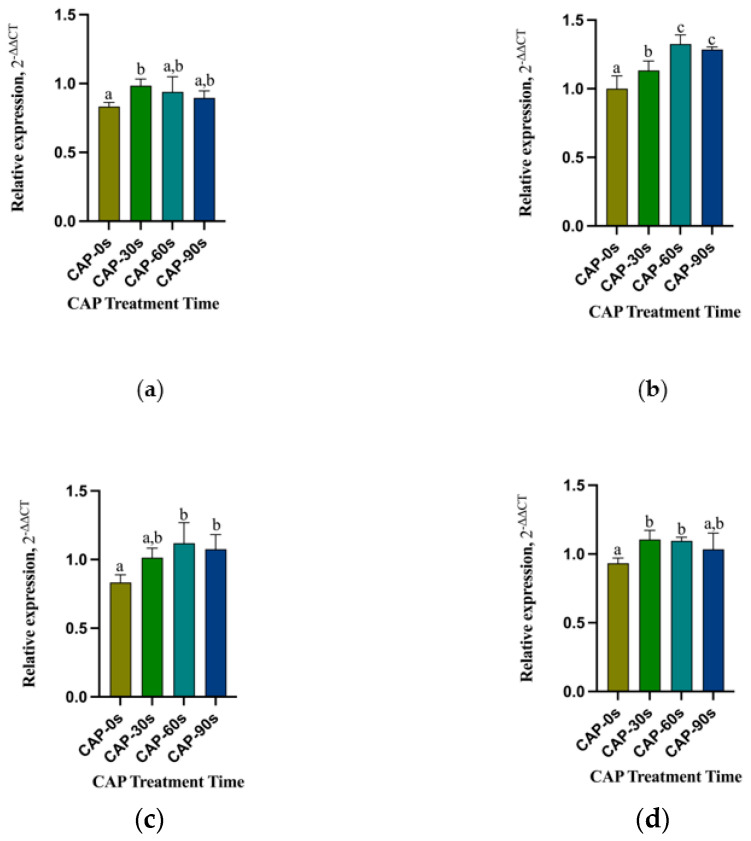
Analyses on the expressions of genes involved in the HGFs’ adhesion on 4Y-PSZ. Analysis of the expression of (**a**) focal adhesion kinase, (**b**) fibronectin, (**c**) integrin β3, and (**d**) vinculin after their culture in real-time PCR for 24 h. Data represent fold changes of the target genes relative to the GAPDH expression and HGFs grown in a control group (100%). The values are presented as means ± SD. Identical letters indicate that the differences between those values have no statistical significance (*p* > 0.05).

**Figure 10:**
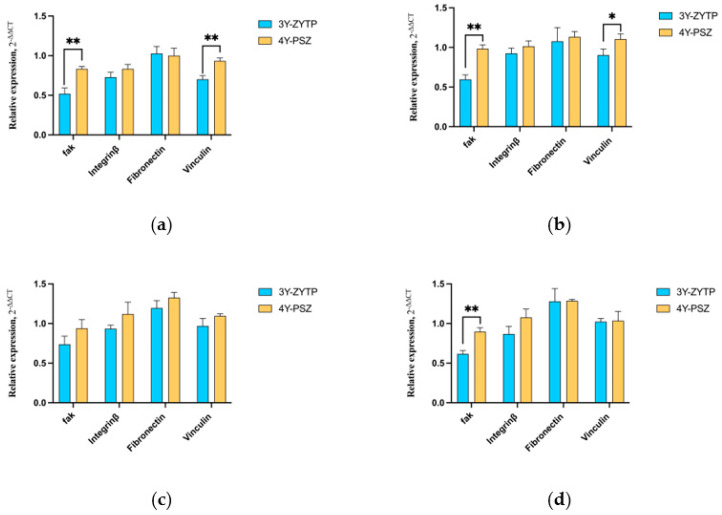
Comparison of expressions of genes involved in the HGFs’ adhesion on 3Y-ZTP and 4Y-PSZ surfaces at the same CAP treatment times (*n* = 12). CAP treatment times: (**a**) 0 s, (**b**) 30 s, (**c**) 60 s, and (**d**) 90 s (*p* > 0.05). (** *p* < 0.01; * *p* < 0.05).

The expression levels of HGFs’ adhesion biomarkers—FAK, FN, VCL, and integrin β3—were upregulated on 4Y-PSZ, as depicted in Figure 9. The expression levels of FAK (CAP-30 s), FN (CAP-30 s, CAP-60 s, CAP-90 s), integrin β3 (CAP-60 s, CAP-90 s), and VCL (CAP-30 s, CAP-60 s) were significantly higher than those of the control group after 24 h of culturing (*p* < 0.05). Additionally, among the three CAP-treated groups, the 4Y-PSZ-60 s group was more favorable for the expression of intracellular FN. At the early stage of cell adhesion, the 4Y-PSZ surface proved more beneficial for the expression of FAK and vinculin (Figure 10a). Following CAP treatment at different durations, 4Y-PSZ also demonstrated enhanced promotion of the expression of adhesion-related genes in HGFs, with FAK (CAP-30 s, CAP-90 s) (*p* < 0.05) and vinculin (CAP-30 s) (*p* < 0.05) showing statistically significant differences (Figure 10b,d).

## 4. Discussion

High-transparency zirconia is an innovative material that combines ample strength with improved transparency, making it a noteworthy choice for abutment applications. Roughness and wettability are the two most critical parameters affecting peri-implant soft tissue integration. Numerous studies have established that hydrophilic abutment surfaces are more conducive to the adhesion and proliferation of connective tissue cells and HGFs on their surfaces [37,38,39,40,41]. However, the impact of different abutment roughness levels on the biological behavior of soft tissue cells remains debated. Compared to roughness, the wettability of the abutment surface has a more pronounced effect on the biological behavior of HGFs [42]. Moreover, increased surface roughness may lead to plaque accumulation, thus elevating the risk of peri-implant disease [43]. Consequently, smooth abutment surfaces with high hydrophilicity are advantageous for the attachment of soft tissue around the abutment [34,35]. According to XPS analysis results, the oxygen content and hydroxyl levels of 4Y-PSZ differed from those of 3Y-ZTP, resulting in superior initial hydrophilicity of 4Y-PSZ. Cytological experiments indicated that 4Y-PSZ, even without CAP treatment, was more conducive to HGFs adhesion and proliferation compared to 3Y-ZTP at various culture times (3 h, 24 h, 48 h, 72 h), and the expression of FAK and vinculin at 24 h was also higher on untreated 4Y-PSZ, corroborating the earlier findings regarding the effects of hydrophilicity on material biocompatibility. These results further affirm that beyond its aesthetic benefits, high-transparency zirconia also offers improved biocompatibility for HGFs.

In terms of modification methods for zirconia abutments, techniques such as sandblasting, acid etching, polishing, and laser treatment risk altering the surface micromorphology and damaging the surface structure of zirconia [19,44,45,46], potentially leading to an inflammatory response in surrounding tissue [47,48]. Ultraviolet irradiation and bionic surface coatings, including HA, collagen, and fibroin, are recognized for their potential to enhance soft tissue formation around zirconia abutments [24,25,49,50]. However, the processing of these methods is complex and time-consuming [51]. 

Cold atmosphere plasma (CAP) offers high efficiency and safety, preserving the surface structure of biomaterials during treatment, thus making it an ideal method for abutment surface modification [52,53]. CAP treatment is widely used in implant and abutment surface modification. Studies have confirmed that CAP treatment can enhance the wettability of the treated surface while maintaining the surface roughness unchanged [52,54,55]. Plasma, rich in free electrons and ions, can generate reactive oxygen species (ROS) and reactive nitrogen species (RNS) on treated surfaces. These include short-lived substances such as hydroxyl radical (OH·), nitric oxide (NO), atomic oxygen, and ozone (O_3_), as well as long-lived substances like hydrogen peroxide (H_2_O_2_), nitrite ion (NO_2_^−^), nitrate ion (NO_3_^−^), and peroxyl groups [56]. ROS generated by CAP treatment can alter the surface elemental composition of materials, consistent with our XPS findings. These results indicate that oxygen and hydroxyl contents on the surface of 4Y-PSZ increase with extended plasma treatment duration, with a trend also observed with 3Y-ZTP. Due to differences in initial elemental compositions, the percentage contents of oxygen and hydroxyl on 4Y-PSZ surfaces were consistently higher than those on 3Y-ZTP under the same CAP treatment conditions. The results of surface wettability for 4Y-PSZ correlate closely with trends in surface oxygen and hydroxyl content, suggesting that the surface oxygen content may be a key factor influencing material hydrophilicity.

The ROS, particularly hydroxyl produced by CAP on zirconia, can adsorb negatively charged proteins, thus enhancing cell attachment [23]. The results in this study suggest that hydroxyl generated by CAP may increase the expression of fibronectin (FN) and integrin β3. The upregulation of these transmembrane proteins could activate the expression of FAK, thereby promoting HGFs adhesion and spreading. As our findings indicate, the amount of cell adhesion, cell surface area, and cell perimeter on 4Y-PSZ surfaces treated with CAP increased. These observations are in line with previous reports of the fibroblast adhesion process on zirconia abutments following plasma treatment [28,57]. Additionally, our findings confirm that CAP treatment of 4Y-PSZ can further enhance HGFs adhesion and proliferation by grafting ROS onto its surface.

Our study revealed that the adhesion and proliferation of HGFs did not increase with extending the CAP treatment time, which diverges from the increasing trend of hydroxyl content on the high-transparency zirconia surface over similar durations. This discrepancy may be attributed to the dose-dependent effects of ROS on cellular behavior [58]. Research on the direct impact of CAP treatment on cells indicates that excessive ROS production can induce apoptosis and death in both normal and tumor cells [59,60,61]. Another potential reason for the inhibitory effects of overdosed CAP on HGFs includes the simultaneous production of UV radiation, thermal radiation, and electromagnetic (EM) waves [62,63]. Among the three CAP-treated groups, the number of adherent fibroblasts was higher after 30 s or 60 s of CAP treatment compared to 90 s (CAP-30 s at 24 h; CAP-60 s at 48 and 72 h). This might be because 4Y-PSZ contains more oxygen, especially hydroxyl radicals, than 3Y-ZTP, and shorter CAP treatment durations achieve more beneficial effects on HGFs adhesion and proliferation. After CAP treatment, the shorter the treatment duration, the more pronounced was the advantage of 4Y-PSZ over 3Y-ZTP in promoting cell adhesion. At 90 s, the effects of the two materials on HGFs were indistinguishable, supporting the conclusion that shorter treatment times enhance the biocompatibility of high-transparency zirconia. This observation also confirms that ROS has a quantifiable relationship with cellular behavior. The optimal CAP treatment duration for 4Y-PSZ is approximately 30 to 60 s.

In this study, we explored the effect of CAP treatment on zirconia with different chemical compositions and its impact on the biological behavior of HGFs. Our findings further elucidate the influence of surface ROS content on cellular behavior and its dose-related nature. However, the optimal treatment conditions for the 3Y-ZTP and 4Y-PSZ zirconia abutments remain undetermined. Additionally, the impact of abutment treatment on bacterial adhesion and proliferation warrants consideration. Further experiments are necessary to determine the effects of CAP treatment on the biological behavior of pathogens in peri-implant diseases, thereby determining optimal operating parameters for obtaining ideal abutment surface treatment results.

## 5. Conclusions

4Y-PSZ zirconia comprises both tetragonal (75%) and cubic (25%) phases, and its initial ROS content is higher than that of 3Y-ZTP zirconia. CAP treatment further increased the ROS content on the surface of 4Y-PSZ zirconia, as evidenced by the enhanced wettability. The modifications in the physical and chemical properties of the 4Y-PSZ zirconia surface induced by CAP treatment were positively correlated with the duration of processing. An appropriate concentration of ROS could enhance the adhesion and proliferation of HGFs on the surface of 4Y-PSZ zirconia by stimulating the gene expression of extracellular matrix proteins and transmembrane proteins. However, the biological behavior of HGFs did not improve proportionally with an increase in ROS content. Therefore, CAP provides optimal conditions for enhancing the biological behavior of HGFs on the 4Y-PSZ zirconia surface when the initial ROS content is higher than that of 3Y-ZTP zirconia. The optimal CAP processing time for 4Y-PSZ zirconia is approximately 30 to 60 s. Thus, this study concludes that high-transparency zirconia, characterized by superior transparency and enhanced biocompatibility with HGFs over shorter CAP processing times, is an ideal material for abutments. Moreover, CAP treatment further improves its biocompatibility with HGFs.

## Figures and Tables

**Table 1:** Primer pairs used in real-time PCR analysis.

Gene	Sequences (5’—3’)	
	Forward	Reverse
Focal adhesion kinase	CTCCTA CTGCCAACCTGGAC	GCCGACTTCCTTCACCATAG
Integrin β3	CAAAGGAACAGCAGAGAAGC	ATTGAGTAAGACAGGTCCATAA
Fibronectin	CGGAGAGACAGGAGGAAATAGCC	TTGCTGCTTGCGGGGCTGTC
Vinculin	CGAATCCCAACCATAAGCAC	CGCACAGTCTCCTTCACAGA
GAPDH	AAGGTCATCCCTGAGCTGAAC	ACGCCTGCTTCACCACCTTCT

**Table 2:** Water contact angle photos of 3Y-ZTP and 4Y-PSZ specimens.

	Group	CAP-0 s	CAP-30 s	CAP-60 s	CAP-90 s
3Y-ZTP	Image	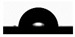	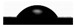	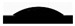	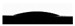
	Water contact angle	83.99 ± 1.22	58.00 ± 1.69	36.00 ± 0.85	19.37 ± 1.36
4Y-PSZ	Image	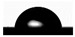	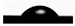	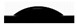	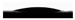
	Water contact angle	81.04 ± 1.55	55.31 ± 1.65	35.04 ± 0.84	17.70 ± 0.78

**Table 3:** Atomic percentages of C and O on 3Y-ZTP and 4Y-PSZ.

		C (%)	O (%)	C/O Radio
CAP-0 s	3Y-ZTP	44.00	35.92	1.22
	4Y-PSZ	36.78	42.63	0.86
CAP-30 s	3Y-ZTP	36.20	43.15	0.84
	4Y-PSZ	38.65	49.23	0.79
CAP-60 s	3Y-ZTP	16.25	55.87	0.29
	4Y-PSZ	11.89	57.8	0.21
CAP-90 s	3Y-ZTP	17.19	57.04	0.30
	4Y-PSZ	13.07	56.97	0.23

**Table 4:** Percentage of OH on 3Y-ZTP and 4Y-PSZ.

		OH (%)
CAP-0 s	3Y-ZTP	22.72
	4Y-PSZ	25.36
CAP-30 s	3Y-ZTP	27.25
	4Y-PSZ	27.75
CAP-60 s	3Y-ZTP	28.30
	4Y-PSZ	28.71
CAP-90 s	3Y-ZTP	30.15
	4Y-PSZ	32.16

## Data Availability

The original contributions presented in the study are included in the article, further inquiries can be directed to the corresponding authors.
